# Text Messaging to Enhance Mindfulness-Based Smoking Cessation Treatment: Program Development Through Qualitative Research

**DOI:** 10.2196/11246

**Published:** 2019-01-07

**Authors:** Claire A Spears, Sharrill A Bell, Charlayne A Scarlett, Natalie K Anderson, Cherell Cottrell-Daniels, Sadaf Lotfalian, Maitreyi Bandlamudi, Amanda Grant, Anna Sigurdardottir, Brittani P Carter, Lorien C Abroms, David W Wetter

**Affiliations:** 1 Division of Health Promotion and Behavior Georgia State University School of Public Health Atlanta, GA United States; 2 Department of Psychology Catholic University of America Washington, DC United States; 3 Prevention and Community Health Milken Institute School of Public Health George Washington University Washington, DC United States; 4 Center for Health Outcomes and Population Equity University of Utah and Huntsman Cancer Institute Salt Lake City, UT United States

**Keywords:** mobile phone, low socioeconomic status, qualitative, short message service text messaging, smoking cessation

## Abstract

**Background:**

Mindfulness-based programs show promise for promoting smoking cessation in diverse populations. Mobile health strategies could increase treatment engagement and in-the-moment support, thus enhancing the effects of mindfulness-based smoking cessation interventions. However, most mobile health programs have been developed without sufficient input from the target population.

**Objective:**

By eliciting input from the target population, predominantly low socioeconomic status (SES) African American adult smokers, throughout the development of an SMS (short message service) text messaging program that teaches mindfulness for smoking cessation, we aimed for the resulting program to be optimally effective and consistent with participants’ needs and preferences.

**Methods:**

Two qualitative studies (N=25) were conducted with predominantly low SES, African American adult smokers. In Study 1 (initial qualitative input; n=15), participants engaged in focus groups to provide suggestions for program development. In Study 2 (abbreviated trial; n=10), participants received a 1-week version of the SMS text messaging program and provided feedback through in-depth interviews.

**Results:**

In Study 1, participants suggested that the SMS text messaging program should be personalized and interactive (ie, involve two-way messaging); provide strategies for coping with cravings and recovering from smoking lapses; involve relatively short, to-the-point messages; and include pictures. In Study 2, participants were highly engaged with the texts, indicated that the program was useful, and provided additional suggestions for improvement.

**Conclusions:**

Eliciting feedback from the target population throughout the intervention development process allowed for iterative revisions to increase feasibility, acceptability, and effectiveness. Overall, SMS text messaging appears to be a feasible, appealing way to provide in-the-moment personalized support and encourage mindfulness among low-income African American smokers.

## Introduction

Tobacco use is the leading cause of preventable morbidity and mortality in the United States [1]. Although most smokers indicate interest in quitting, the rates of successful smoking cessation are quite low (eg, based on 2015 data, only 7.4% of adult smokers had quit in the past year [2]). Adults with low socioeconomic status (SES) exhibit disproportionately high rates of smoking, often have greater difficulty quitting, and consequently experience profound tobacco-related health disparities [2-6]. Compared to other racial or ethnic groups, African Americans tend to have greater difficulty quitting smoking and higher incidence and mortality rates for diseases associated with smoking [2-4,7,8]. There is an urgent need to develop accessible, evidence-based smoking cessation interventions targeting disparity populations, including low SES and African American smokers.

Mindfulness is defined as purposeful, present-focused attention with an attitude of acceptance and nonjudgment [9-11]. Mindfulness-based interventions show promise for improving smoking cessation outcomes in diverse populations [12-17]. Moreover, mindfulness may be particularly beneficial in low SES and racially or ethnically diverse populations. Practicing a nonjudgmental, compassionate attitude toward oneself could be especially useful for people from marginalized backgrounds [18], and mindfulness practice (eg, mindfully focusing on one’s breath) does not require a high level of education or resources. We found that low SES, predominantly African American adults perceived the ability to practice mindfulness on their own (regardless of availability of external resources) as empowering and beneficial to both mental and physical health [19]. Regardless of the target population, between-session mindfulness practice (eg, sitting meditation, gentle yoga, mindful awareness of breath) is thought to be integral in producing benefits [10]. However, participants do not always practice mindfulness in their daily lives [20].

Mobile health (mHealth) technology presents unique opportunities for mindfulness research and intervention, and cell phone use has become ubiquitous in the United States [21]. Between-session short message service (SMS) text messaging might be an effective means to encourage participants to use mindfulness techniques in moments when they need them most, thus enhancing treatment effectiveness. Furthermore, data suggest that adults from racial or ethnic minority backgrounds and those with lower education tend to text more frequently than Caucasians and those with higher education [22]. Thus, SMS text messaging might be an appropriate way to target smoking cessation in low SES and African American smokers.

There is substantial evidence supporting the efficacy of SMS text message-based smoking cessation interventions (for reviews, see [23,24,25]); however, these interventions have not focused on mindfulness. Thus, the current qualitative studies were designed to inform the development of SMS text messages to be used in a mindfulness-based smoking cessation intervention. Participants from low SES backgrounds were targeted with the goal of creating a SMS text messaging program that is engaging, acceptable, and optimally effective in this population. Researchers have highlighted the critical need to elicit feedback from the target population at the outset and throughout the process of developing mHealth interventions [26,27]. Unfortunately, mHealth programs are often developed with insufficient input from the target population [28] or lack of attention to evidence-based strategies [29].

Abroms et al [26] recommended steps for developing and pretesting text messaging-based health promotion programs were followed. First, we defined the population of interest as low SES adult smokers (with a large proportion of African Americans represented given the racial distribution of the cities where the intervention was developed) and conducted focus groups (Study 1) to inform the initial development of the intervention. Study 1 assessed participants’ preferences regarding timing and content of SMS text messages. Next, we designed the initial SMS text message library, with a focus on evidence-based mindfulness and cognitive-behavioral strategies for quitting smoking. SMS text messages were intended to remind participants of specific strategies in times of need, as well as to encourage daily mindfulness practice (which could increase facility of mindfulness techniques in high-risk and other situations). We developed a brief, 1-week version of the program to be pretested with a separate group of participants (Study 2) and then used participant feedback to further improve the program. A longer version of the program will then be evaluated in a randomized controlled trial, with continued revisions as needed. Through gathering in-depth qualitative input from the target population throughout intervention development, our overarching aim is for the resulting program to be optimally effective and consistent with participants’ needs and preferences.

## Methods

### Study 1: Initial Qualitative Input

#### Participants

Participants were recruited from the Washington, DC metropolitan area through flyers at local community health centers and community centers and shelters and through Craigslist. The inclusion criteria were as follows: age 18-65 years (individuals aged >65 years were excluded given lower use of texting in this population [22]); current smoker with history of ≥5 cigarettes per day for the past year (verified in-person with expired carbon monoxide, CO, ≥6ppm); motivated to quit within the next 30 days; valid home address in the greater DC area; functioning telephone number; can speak, read, and write in English; and marginal or adequate health literacy (determined using the Rapid Estimate of Adult Literacy in Medicine [30]). The exclusion criteria were as follows: contraindication for nicotine patch, active substance abuse or dependence, regular use of tobacco products other than cigarettes, current use of tobacco cessation medications, pregnancy or lactation, or a household member enrolled in the study. These inclusion and exclusion criteria were chosen in order to maintain consistency with eligibility criteria for the later randomized controlled trial of the SMS text messaging intervention to be developed.

#### Procedures

After completing eligibility screening and providing informed consent, participants completed a background questionnaire to indicate demographic information and mobile phone usage. Focus groups were approximately 90 minutes each, and the facilitator asked participants about their level of interest in SMS text messaging to help them quit smoking; suggestions for the content of messages (including reactions to sample SMS text messages); preferences for structure and timing of messages; and other ideas for making the program more helpful and user-friendly.

Group sessions were audio-recorded with a digital voice recorder and transcribed verbatim. Transcripts were managed and coded using NVivo 10 software (QSR International). The data coding and analysis followed both inductive and deductive approaches [31,32]. The first author (who facilitated the focus groups) and 2 separate coders (trained research team members) each reviewed the transcripts to develop an initial set of themes from the interview topics and the conceptual framework, with additional codes identified from concepts that emerged from the data. After developing the initial coding scheme, the 2 coders each independently coded one focus group and met as a group with the first author to review inconsistencies and further refine the coding scheme. The remaining transcripts were independently coded, with regular meetings to discuss the coding process and discrepancies, maintain consistency over time, and refine the coding scheme as needed. Discrepancies were resolved through group discussion, with final decisions made by the first author.

### Study 2: Qualitative Feedback After Text Messaging Trial

#### Participants

Recruitment and inclusion and exclusion criteria were identical to those in Study 1, with 2 exceptions. Study 2 was conducted in the Atlanta, Georgia metropolitan area, and eligible participants needed to have a cell phone with SMS text messaging capability and an unlimited SMS text message plan.

#### Procedures

After completing informed consent, participants completed a brief questionnaire inquiring about demographic information and mobile phone usage. Because participants were not necessarily expected to have experience with mindfulness, research staff provided a definition of mindfulness, discussed ways to practice mindfulness in daily life, and informed participants that the SMS text messages would involve mindfulness. Participants provided the researchers with their mobile phone numbers in order to receive SMS text messages over the following week. As participants in the later intervention study would receive SMS text messages for 7 weeks, each of the 7 days in this pilot study was designed to mimic each week of the planned intervention study (ie, on day 3 of this pilot, participants received the same number and types of messages that they would receive during week 3 of the intervention; this allowed us to collect information on various types of messages that would be sent at different stages of the quitting process). The research team explained this to participants and asked them to imagine that Day 5 was their quit day (corresponding to week 5 of the intervention). Participants received 2 messages on Day 1, 3 on Day 2, 4 on Day 3, 5 on Day 4, 6 on Day 5, 4 on Day 6, and 3 on Day 7. In addition, participants could text CRAVE, STRESS, or SLIP at any point during the week to receive additional SMS text message support for coping with cravings, stress, or smoking lapses, respectively.

Mindfulness techniques incorporated into the 1-week period were mindful breathing, mindful eating, mindful stretching, and mindful awareness of body sensations (including experiences of craving). These are core practices of Mindfulness-Based Addiction Treatment for smoking cessation [17] and were adapted as brief, in-the-moment practices (eg, “Stand up and take a deep breath. Move your arms slowly in circles and then stretch your whole body from side to side, noticing how you feel”). Participants were asked to keep a log of their responses to and suggestions for improving the SMS text messages and to bring this log to their next appointment to facilitate the recall of their experiences during the interview.

After the 1-week period, participants returned for an in-person individual interview, which was audio-recorded and transcribed verbatim. The interview guide inquired about participants’ experiences receiving the SMS text messages, aspects that were helpful and not helpful, suggestions for improving the SMS text messages, suggestions regarding the number and timing of messages, experiences using (or not using) the keywords, and any concerns about receiving and sending SMS text messages for smoking cessation (including any privacy concerns). The general analytic approach was identical to that in Study 1.

## Results

### Study 1

#### Participant Characteristics

A total of 15 adult smokers participated in the focus groups (3 groups of 4 participants each and 1 group of 3 participants). The majority of participants were women (12/15, 80%), African American (13/15, 87%), and reported a total annual income of <$18,000 (9/15, 60%). The mean age was 44.3 (SD 11.6) years. Participants smoked 14.3 cigarettes per day on average (SD 9.8) and most (10/15, 67%) reported smoking within 30 minutes of waking. All participants indicated that they currently owned a mobile phone with an unlimited SMS text message plan. On average, participants indicated that they receive 14.9 (SD 8.7) texts per day and send 13.7 (SD 9.1) texts per day.

#### Qualitative Results

Major themes were the perceived acceptability or helpfulness of SMS text messages for smoking cessation; concerns about text messaging; suggestions about the content of messages; and suggestions about the format of messages.

#### Perceived Acceptability or Helpfulness of Text Messages for Smoking Cessation

Overall, participants were open to the idea of SMS text messaging for smoking cessation, and most were enthusiastic. Some participants were skeptical that texts would help them quit in the absence of other support but believed that SMS text messages in addition to in-person treatment and nicotine patches would be helpful. Participants thought texts would be helpful because “it gives you something else to do instead of smoking a cigarette” and “you could be ready to light a cigarette, and see the text message…and put [the cigarette] down.” They noted that texts could help remind them of their goal to quit smoking; for example: “I think it’s a positive thing to get text messages so my awareness stay up. A lot of times I say I’ll quit smoking, and I forget that I said that.” Participants also liked the perceived privacy of text messaging, discerning this form of communication to be discrete; for example: “I was thinking the whole texting is good too, ‘cause it’s private….It’s between you and what’s on your phone. No one has to hear your response.”

#### Concerns About Text Messaging for Smoking Cessation

##### Concerns About a One-Size-Fits-All Approach

The most common concern was that SMS text messaging might work for some people and not others (“different strokes for different folks”), mostly based on personal SMS text messaging habits. One person said, “I don’t even read my texts half the time.” Another suggested that an SMS text messaging program “is for someone who really uses their text messages.”

##### Concerns About Too Many Texts

Participants noted that too many texts and requests to answer questions via text could be “annoying,” arrive at inconvenient times, or be ignored by users (eg, “It’s going to start bothering people;” “If I had to do a lot of texting and a lot of questions I’d have to answer, I’d be a little frustrated”). One person noted, “I wouldn’t answer a lot of [texts]. I would probably block it out…if I’m at a party and I’m drinking.”

#### Suggestions About the Content of Text Messages

Regarding the content of SMS text messages, common themes included suggestions for personalization; strategies for managing cravings; encouragement for coping with lapses; and visual messages.

##### Personalization

A common theme was that the program be highly personalized. For example, one participant indicated, “It has to be personalized to their life, day by day.” Participants suggested personalization in terms of the content of text messages (eg, reminders for personal reasons for quitting: “Maybe in the beginning, you list why you want to do the program in the first place and it will periodically text you…‘Remember, you wrote this down’”). They also suggested personalization of the text schedule (eg, “individually set your hours”).

##### Specific Strategies for Managing Cravings

Participants were interested in receiving texts with specific strategies to cope with cravings (eg, “I want to hear a way to cope with [cravings] and something else that I could do instead of smoking a cigarette to make the craving go away”). Another participant noted, “For me, if I’m craving…I have a really hard time thinking. So, [the text could give] a very solid list of things I can do to make the craving go away, and I don’t have to think of it myself.” Another said, “I like the suggestions. You know, try to do something, play a game, take your mind off it, call somebody, I like that.”

##### Encouragement for Coping with Lapses

Participants noted that they would appreciate encouragement to forgive themselves and get back on track after smoking lapses. One participant suggested messages like “Nobody is perfect. It’s hard to get off cigarettes….Don’t beat yourself up! You made a mistake but you’ve got more lives in this game!” Another indicated that it would be helpful to hear “Brush yourself off, get back on the bike, keep going, try again. Don’t beat yourself up. Alright, you messed up, but try it again. See what happens next time.”

##### Visual Text Messages

In addition to traditional SMS text messages, participants suggested including images. One participant noted, “I like the text pictures…that would just stick in my mind. But words, I have to read it for a while. [Pictures] that will just pop out at you would be nice. And it would do more good than reading a message.” Another said, “If I’m stressed, I don’t know what would calm me down. Something visual though, something I could look at. Something positive…something relaxing.” While some suggested positive images (eg, “flowers,” “something funny”), others suggested graphic images of health effects of smoking (eg, “hole in someone’s throat from smoking”).

#### Suggestions About Format of Messages

Regarding SMS text message format, participants suggested that the messages be as short as possible. They also suggested interactive two-way messaging and frequent messaging.

##### Interactive Text Messages

Participants indicated that in addition to receiving texts, they wanted to be able to initiate and respond to texts. Example quotes included “If you get a text message, then you have to somehow respond to it, or log something so you feel like you are invested;” and “I think if every time we want to smoke a cigarette, we should grab the phone and text you.” Another participant said, “If you’re going to try to help people through text messages, let them hit you when they need you. You have to be available when I need you…Like right now I’m thinking about smoking a cigarette…Let me pick up this phone, and see what [the text] could say to me. I’m texting you.”

##### Number and Timing of Text Messages

Participants overwhelmingly suggested that SMS text messages be sent frequently (eg, “every hour or maybe every half hour;” or “at least 4 to 5 times a day”). Participants indicated, “It has to be frequent and it has to be repetitious…if you’re serious about quitting,” and “If you get enough of them throughout the day, it’ll make you think twice before you light up another cigarette.” Participants preferred a graded (vs fixed) message schedule, in which they would receive more frequent SMS text messages on and around their quit date.

#### Initial Design of Text Message Library

The SMS text messaging intervention was designed to encourage mindfulness-based and cognitive-behavioral strategies for smoking cessation. Based on suggestions from focus groups, SMS text messages were designed to be personalized (eg, participants’ first names were used, and they were reminded of their personal reasons for quitting and the amount of money they would save after various time periods of quitting) and interactive. Participants were asked questions (eg, “There are people, places, and things that make you want to smoke. What are your top 3 triggers to smoke?” “Good morning, David! Would you like to try a mindfulness exercise?,” and “Have you smoked today?”), with automated responses based on participants’ replies. Participants could also initiate texts by sending keywords CRAVE, STRESS, or SLIP for support. Texts after participants’ smoking lapses promoted self-compassion and encouragement for getting back on track. Picture messages were incorporated (eg, nature images with captions like “Breathe” and “Today is a new day”). A graded message schedule was used, such that the frequency of texts increased around the quit date and then gradually reduced after the quit date.

### Study 2

#### Participant Characteristics

A total of 10 adult smokers (5 women and 5 men; separate from those in Study 1) participated in Study 2. The majority (8/10, 80%) of smokers were African American individuals; 1 was a Caucasian, 1 reported ≥1 race, and 1 was a Hispanic person. Most (7/10, 70%) participants reported a total annual income of <$18,000. The mean age was 44.9 (SD 9.7) years. Participants smoked 21.3 (SD 6.9) cigarettes per day on average and most (8/10, 80%) reported smoking within 30 minutes of waking. Excluding 1 participant who indicated sending and receiving 800 texts per day, participants indicated that they receive 29.1 (SD 19.7) and send 18.2 (SD 10.2) texts per day on average.

#### Engagement With Text Messages

All participants answered at least some of the questions that were asked of them via text. All participants texted the system back (at least once) when they were not specifically prompted to answer a question (eg, by responding “thank you” after messages, although they were not specifically asked to do so). The number of these texts ranged from 1 to 24 (mean 11.6, SD 8.5). Of the 10 participants, 7 texted a keyword (CRAVE, STRESS, or SLIP) at least once. Among the participants who used keywords, the number of keyword texts ranged from 1 to 11 (mean 3.6, SD 3.4). Accordingly, 6 participants texted CRAVE, 2 texted STRESS, and 4 texted SLIP.

Overall, participants’ SMS text message responses during the 1-week period indicated that the texts were perceived as helpful. For example, when a text suggested taking a walk to relax, 1 man texted, “I wanted a cigarette this morning so instead of smoking I walked to the store it was a good walk it made me forget about the cigarette.” When encouraged to seek support from friends and family, 1 man responded, “I have talked about it with my sister she told me don't be hard on myself just brush myself off and start over again so she is being very supportive.” Several participants responded to text questions to indicate their smoking triggers (eg, “stress,” “people,” “being angry,” “after I eat”), strategies that might work for them to cope with cravings (eg, “toothpicks,” “brushing teeth,” “gum”), and ways that they will reward themselves when they quit (eg, “a slice of New York cheesecake,” “buy a new pair of winter boots,” “go to the movies”). Participants often texted positive responses including “Thank you for that inspiring message” and “That is a real motivational thing to say to myself.” Participants also indicated positive experiences with the mindfulness exercises (eg, by indicating that they were “peaceful” and “relaxing;” saying “I really needed that, thanks”).

#### Qualitative Results

Major themes were the perceived acceptability or helpfulness of SMS text messages for smoking cessation, most helpful aspects of texts, and suggestions for improvement.

#### Perceived Acceptability or Helpfulness of Text Messages for Smoking Cessation

Overall, participants noted positive experiences receiving the SMS text messages, and several indicated that the texts helped them to quit or reduce their smoking over the week-long period (eg, “I like them because they were straight to the point. They helped me quit smoking;” “I cut back a whole lot”). Examples of participants using the texts to avoid or reduce their smoking are “I just grabbed my phone instead of a cigarette” and “It does help when the text message comes, you know? Couple times I got a text and dumped the whole cigarette out the window.”

Regarding the most helpful aspects of the SMS text messaging program, participants commonly noted the positive, encouraging tone; mindfulness; social support; strategies for avoiding or coping with triggers; picture messages; the opportunity to text keywords; and personalization. Participants dismissed any concerns about privacy (eg, other people seeing their texts or giving their phone number to the research team).

#### Most Helpful Aspects of Text Message Program

##### Positive Tone

Participants appreciated the positive, encouraging tone. One participant indicated, “When you’re on these streets, there's so much negative and then when you hear something positive through a phone, it just makes you wanna do good.” Another said, “Basically [the text] was like, ‘Mistakes happen, you know? But keep pushing and try to get back on track.’ That was helpful.”

##### Mindfulness

Participants found value in messages encouraging mindfulness. One participant said, “The mindful text message was pretty helpful. Take a minute and just be in that minute right there. I did that when I was driving one day and just took a deep breath, not smoking a cigarette. It makes you feel a little good about yourself right quick.” Another said, “I loved it. I’d only read about mindfulness as far as mindful eating, but I never really considered applying it to my entire life, and it’s really simple. You just stop take a deep breath, stretch and it really does help. Something so easy can be so helpful.” Another said that when stressed, the most helpful message would be “that mindful one. Take a moment, take a breath… stretch.” Overall, participants found it relatively easy to engage in these types of mindfulness practices in the moment (mindful breathing was most often mentioned in this context). Several appreciated learning to focus on the present (eg, “don’t worry about the past or the future, just be in this moment”) and described incorporating regular mindfulness practice into their daily lives (eg, “Every day. I do about 3-4 minutes”).

##### Social Support

Although participants knew that the program was automated, they often described a sense of social support from the SMS text messages. Example quotes were “It’s like having a friend who texts you when you are feeling stressed or having a feeling like you want to smoke” and “It’s like having a little quitting coach that encouraged you along and helps you out when you need help.”

##### Strategies for Avoiding or Coping with Triggers

Participants noted that texts encouraging them to remove triggers (“helped me get rid of my ashtray, cigarette lighters, and all of that”) or use specific strategies to cope with cravings were useful. One participant said, “[The text messages] were very helpful… the substitute, you know, gum or candy, or meditating like I was saying. Finding someone to call if I'm craving.”

##### Picture Text Messages

In general, people liked the picture messages (though some participants suggested including graphic images that were not included in the program; see “Tough Love and Graphic Warnings” below). One person said, “[The pictures] motivated me and gave me a reason to stop smoking.” Picture messages were described as “peaceful” and “inspirational.” One participant noted that he was charged extra by his wireless provider for downloading pictures, but he decided to download them anyway because they were helpful.

##### Keywords

Participants appreciated the opportunity to initiate interaction by texting CRAVE, STRESS, or SLIP. One participant said, “It was really helpful, and being able to say, ‘I want to smoke, somebody help me!’” Another said, “It's very helpful to have somebody you can rely on. You know, if you’re craving, you put ‘crave,’ it's gonna send a message or picture or exercise, or something to do… tell you to do stuff except smoking.” Participants noted the positive tone of the responses to keywords: “When I did text SLIP or CRAVE it didn’t make me feel bad. It wasn’t condemning. It just gave me more encouragement.”

##### Personalization

Participants appreciated the texts reminding them of how much money they would save when they quit smoking (based on their individual smoking habits and price per pack) and personal reasons for quitting. One participant noted, “It gave me a reason to want to stop smoking… about my family, I want to see my grandkids grow up, I want to go on jogs, I don’t want cancer.”

#### Suggestions for Improvement

Some participants indicated that there were not enough SMS text messages, and some said that they came at inconvenient times. Suggestions for improvement included sending more frequent SMS text messages; more personalization; incorporating religion or spirituality; reminding participants of the CRAVE, STRESS, and SLIP keywords; and incorporating “tough love.”

##### More Frequent Text Messages

The most common suggestion was to include more messages, especially around the quit day. Example quotes were “I would have liked to receive more messages, because it just made me feel better about not smoking” and “I would love to have more of them, because I still have them and I’m going to go over them.” Participants suggested sending texts every 15, 30, or 60 minutes, highlighting the need for constant reminders throughout the day (eg, “it will keep reminding me… because when you smoke, you don’t just smoke one now, and one every two to three hours, some people smoke back to back to back.”).

##### More Personalization

Participants often indicated that tailored timing of messages to their personal schedule would be useful (eg, timing around work schedules and high-risk times of day). They also suggested sending personally motivating pictures (eg, “Personal pictures. That is gonna really make it hit home” and “I would have liked to see pictures of my kids.”).

##### Incorporating Religion or Spirituality

Several participants mentioned religion or spirituality and suggested including messages like “Pray,” “Let go and let God,” and “Have faith in God” or incorporating “scriptures from the Bible.”

##### Reminders of Keywords

Some participants indicated forgetting about the keywords, what the keywords were (CRAVE, STRESS, SLIP) or what they meant. For example, 1 participant indicated that he would be more likely to use them if the program would “repeat [the keywords] over and over again, and explain to me what they mean.”

##### “Tough Love” and “Scare Tactics”

Some participants indicated that the messages should be more direct (eg, “Just don’t smoke”), “tougher,” or include “tough love, not soft love.” One person suggested, “Be hard on me.” Some participants suggested including graphic images to elicit fear. One participant explained, “Pictures of people who have holes in their necks… those pictures are scary, but they have to make the public aware…. Scare tactics are a good thing.” Another suggested sending a “picture of a burned-up lung.”

## Discussion

### Principal Findings

Two qualitative studies with predominantly low-income African American adult smokers provided in-depth information about how and why SMS text messaging might be useful for smoking cessation, with specific suggestions for improving interventions for this population. Based on suggestions from Study 1 (initial qualitative input), the SMS text messaging program was designed to be personalized and interactive; provide mindfulness-based and cognitive-behavioral strategies for coping with cravings and recovering from lapses; involve relatively short, to-the-point messages; and include pictures. In Study 2 (abbreviated trial), participants who received a 1-week version of the program were highly engaged with the texts, indicated that the program was useful, and provided additional suggestions for improvement. Eliciting feedback from the target population throughout the intervention development process has allowed us to iteratively revise the program to increase feasibility, acceptability, and effectiveness. Overall, SMS text messaging appears to be a feasible, appealing way to provide in-the-moment personalized support and encourage mindfulness among low-income African American smokers.

### Comparison With Prior Work

Several themes fit well with findings from other qualitative studies of SMS text messaging for smoking cessation. For example, the personalization of SMS text messages was deemed to be important among young adult smokers [33], pregnant smokers [34], and a clinical sample of adult smokers recruited through an emergency department [35]. Quotations from the current sample echo those from smokers in other studies suggesting the importance of tailoring messages to individual schedules, preferences, and motivations [33,35]. Giving participants more choice in the content and timing of messages may enhance their sense of control [33] and reduce the likelihood that people become frustrated or inconvenienced by the texts. A related issue is the potential for SMS text messages to inadvertently stimulate craving for cigarettes [34,36,37]. Although this did not come up in this study, allowing participants to control the number and timing of SMS text messages could help prevent such unintended effects. Messages could also be tailored to personal preferences regarding message tone (eg, positive encouragement vs “tough love”). As in this study, some but not all participants in Bock et al’s [33] study advocated for the use of “scare tactics.”

Notably, although participants were aware that the SMS text messaging system was automated, many noted the social support and accountability that the texts offered, much like a “friend” or “coach.” This is a common theme across SMS text messaging programs, which have been described as “somebody holding your hand” [37] or as though “they were really on my side” [35]. Participants often attribute human characteristics to digital programs, noting a sense of accountability and feeling “shame,” “guilt,” or “a need to obtain ‘forgiveness’” after lapsing [36]. Greater personalization of texts may foster the sense of a “relationship,” and research is needed to determine optimal ways to promote adaptive “relationships” with mHealth programs.

Although there are a number of commonalties between this study and extant research on SMS text messaging for smoking cessation, several unique aspects are noted. First, our program places central focus on encouraging mindfulness during the process of quitting smoking. Overall, the concept of mindfulness and related SMS text messages were well received in this sample of low-income, predominantly African American adults. Given that the majority of research on mindfulness has been conducted with higher income, primarily Caucasian samples, this study adds to the growing literature on perceived acceptability and utility of mindfulness interventions in lower income, African American samples [19,38,39]. Participants in mindfulness treatment studies often do not practice mindfulness on their own as much as instructed [20]. For example, rates of between-session mindfulness practice were relatively low in a recent trial of Mindfulness-Based Addiction Treatment for adult smokers [17]. SMS text messaging could be a novel way to promote daily mindfulness practice during the process of quitting, which might enhance smoking cessation outcomes.

Second, participants noted that religious and spiritual factors motivate them to quit smoking, and we added SMS text messages to address participants’ suggestion to include spiritualty. Incorporating spirituality and religious coping, as well as integrating with faith-based organizations, have been identified as culturally important factors for tailoring health promotion interventions in African American communities [40,41]. In fact, religious coping and religious support may help to buffer the effects of stress on tobacco use among African Americans [42]. However, appeals to organized religion may be off-putting for people without religious affiliation, and a more general focus on spirituality in the context of health promotion may be more broadly accepted by participants [43]. Thus, we created messages incorporating spirituality without reference to any specific religion (eg, “Look to your spiritual beliefs for comfort and strength. You might pray or read/listen to something that is inspirational to you”). Such messages might also fit well in the context of mindfulness treatment. For example, in a qualitative study of mindfulness among low-income, primarily African American adults, participants noted practicing mindfulness through religious or spiritual experiences (eg, while praying, listening to religious music, or reading sacred texts [19]). Although mindfulness-based smoking cessation treatment is typically taught in a secular context, it may be worthwhile to encourage individuals to incorporate spirituality in any way that is helpful.

Third, the inclusion of pictures is a unique feature that was added explicitly based on participants’ suggestions. Texts containing pictures may be more vivid and memorable than those containing only words. Moreover, pictures that do not require extensive reading may be especially appealing and effective for adults with lower levels of education and reading ability. More research is needed to continue to develop and evaluate pictures that are especially motivating for smoking cessation. As participants in this study suggested, SMS text messaging programs might even collect personally motivating pictures from participants and send them back at high-risk times.

### Limitations

The current studies are limited by small samples of predominantly low-income African American smokers living in urban areas, and larger-scale trials are needed to evaluate the efficacy of SMS text messaging to enhance mindfulness-based smoking cessation treatment. Small sample sizes are typical of qualitative research, and the current research focuses on an underserved population at risk for tobacco-related health disparities. However, given our focus on this specific population and additional exclusion criteria (eg, excluding those with active co-occurring substance abuse or dependence), results may not generalize to other populations. Furthermore, larger studies that experimentally test the efficacy of various approaches (eg, whether greater personalization leads to better engagement and quit rates) are needed to evaluate whether certain treatment components work better than others.

### Conclusions

Based on iterative user feedback, SMS text messaging appears to be an acceptable intervention for smoking cessation among predominantly low-income African American adults. Suggestions for SMS text messaging smoking cessation programs in this population include sending texts that are short, to the point, and interactive (with reminders of available keywords); providing in-the-moment strategies for coping with cravings; including visual messages; incorporating spirituality; and using personalization (eg, reminders of personal motivation for quitting and tailoring timing and frequency of messages). Although some of these strategies have been discussed in extant studies (eg, importance of personalization), others (particularly the use of visual messages and spirituality) will uniquely inform our future work on SMS text messaging with low-income African American smokers. Participants disagreed on whether the program should include “scare tactics,” and based on mixed evidence and potential negative consequences such as increased anxiety leading to stronger urges to smoke [44], as well as lower compatibility with mindfulness-based approaches, we have not included scare tactics in our current program. However, many of our participants’ suggestions could be relatively easily integrated into existing SMS text messaging programs and inform SMS text messaging as an adjunct to evidence-based in-person services (eg, cognitive behavioral treatment [45]).

SMS text messaging may have great potential for encouraging mindfulness and other strategies for smoking cessation, in addition to fostering a sense of social support. This mode of intervention, which is relatively low cost and available to participants 24/7, could be particularly useful for underserved populations with lower access to evidence-based smoking cessation services. Continued research is needed to further improve, evaluate, and (if efficacious) more widely disseminate mindfulness-based SMS text messaging interventions for smoking cessation.

## Abbreviations

mHealth: mobile health
SES: socioeconomic status
SMS: short message service
